# Purification of Fibroblasts From the Spiral Ganglion

**DOI:** 10.3389/fneur.2022.877342

**Published:** 2022-04-15

**Authors:** Annett Anacker, Karl-Heinz Esser, Thomas Lenarz, Gerrit Paasche

**Affiliations:** ^1^Department of Otolaryngology, Hannover Medical School, Hannover, Germany; ^2^Stiftung Tierärztliche Hochschule Hannover, Hannover, Germany; ^3^Cluster of Excellence Hearing4all, Hannover Medical School, Hannover, Germany

**Keywords:** inner ear, spiral ganglion cells, cochlear fibroblasts, Thy1, vimentin, cell sorting

## Abstract

Using cultures of freshly isolated spiral ganglion cells (SGC) is common to investigate the effect of substances on spiral ganglion neurons (SGN) *in vitro*. As these cultures contain more cell types than just neurons, and it might be beneficial to have cochlear fibroblasts available to further investigate approaches to reduce the growth of fibrous tissue around the electrode array after cochlear implantation, we aimed at the purification of fibroblasts from the spiral ganglion in the current study. Subcultivation of the primary SGC culture removed the neurons from the culture and increased the fibroblast to glial cell ratio in the preparations, which was revealed by staining for vimentin, the S100B-protein, and the 200-kD neurofilament. We performed direct immunolabeling for the Thy1-glycoprotein and the p75NGFR-enabled fluorescence-based cell sorting. This procedure resulted in a cell culture of cochlear fibroblasts with a purity of more than 99%. The received fibroblasts can be subcultivated for up to 10 passages before proliferation rates drop. Additionally, 80% of the cells survived the first attempt of cryopreservation and exhibited a fibroblast-specific morphology. Using the described approach provides a purified preparation of cochlear fibroblasts, which can now be used *in vitro* for further investigations.

## Introduction

Fibroblasts are the main cells of the loose fibrous tissue and are responsible for the formation of the extracellular matrix (1). Besides fibroblasts, fibrous tissue contains also other cells such as lymphocytes, granulocytes, macrophages, and mast cells, which are a part of the immune defense. The morphology of the fibroblasts can be variable and appears to be tissue-specific (1). Fibroblasts are involved in wound healing and encapsulation of foreign bodies (2, 3). In the cochlea, this is of special importance in connection to cochlear implantation. After the insertion of the cochlear implant (CI) electrode array into scala tympani, an increase in electrical impedance at the stimulating contacts can be observed (4, 5). Even though not all authors agree, this was shown to be connected to the formation of fibrous tissue around the electrode array (6, 7).

Potentially, there are at least three possible sources where fibroblasts could be recruited for this process. The first option is that the fibroblasts are derived from pieces of tissue (muscle or fascia) that are typically used to seal the entrance of the electrode array into the cochlea, which can be a cochleostomy or the round window (8). Indication for this was found in earlier studies showing that the increase in impedance starts at the basal electrodes (5). But this study also showed that the impedance increase can be higher in the apical contacts than in the middle contacts. This might indicate that there are additionally other sources of the fibroblasts. As the electrode arrays have to be inserted into the spiral of the scala tympani, they will most likely touch the cochlear walls during insertion or when taking its final position. This might, depending on the electrode type (straight or perimodiolar) and insertion technique, injure either the lateral wall or/and the modiolar wall close to Rosenthal's canal and could include bleeding, which is known to enhance tissue formation (9). These regions are considered the other two possible sources of fibroblasts.

Despite huge efforts to reduce tissue growth after cochlear implantation (10), this problem is not solved yet and needs further attention. To facilitate further *in vitro* tests in this direction, organ-specific cell cultures might be helpful. There is one report on an immortalized fibroblast cell line originating from type I fibrocytes of the spiral ligament (11). The authors considered this cell line a research tool for investigating homeostasis and inflammation in the ear and it was used in several reports in this context. According to Yian et al. (11), type I fibrocytes can mainly be found next to stria vascularis, far away from scala tympani.

In our lab, the preparation of spiral ganglion cells (SGC) was established for many years to investigate the effects of drugs and electrical stimulation on spiral ganglion neurons (SGN) (12, 13). But this is a mixed culture containing more of other cells than neurons. Besides neurons, the spiral ganglion contains mainly glial cells of the peripheral nervous system and endoneural fibroblasts, which are embedded in the endoneural fibrous tissue. The spiral ganglion is surrounded by a fibrous capsule, which extends into the epi- and perineurium of the nerve fibers (14). Therefore, it is to be expected that at least glial cells and fibroblasts are part of the dissociated SGC culture. Enrichment and purification of Schwann cells from these cultures using either different adhesion properties to surfaces (15) or an immune-magnetic purification procedure (16) was described, but there was no purification of fibroblasts. Both authors used the p75NGF-receptor staining to identify the Schwann cells of the spiral ganglion, as described by Whitlon et al. (17).

The aim of the current study was to purify cochlear fibroblasts from the existing mixed culture of SGC and make them available for other investigations *in vitro*.

## Materials and Methods

### Ethics Statement

All experiments were carried out according to the German “Law on Protecting Animals” (§4) and with the European Directive 2010/63/EU for the protection of animals used for experimental purposes. These experiments were registered (2013/44 and TVT-2017-V-95) with the local authorities.

### Animals

Sprague–Dawley rats (postnatal days 2–4) of both sexes were used for the experiments with one exception (postnatal day 1). Animals were kept at room temperature with 50–60% relative humidity. They had access to food and water *ad libitum*. Day–night cycle was automatically regulated with 12 h each. Rat pups were separated from their mothers about 30 min before cell preparation and kept in groups at 37°C.

### Preparation of Spiral Ganglion Cells

Spiral ganglion cells were prepared as described by Wefstaedt et al. (12). Briefly, the rats were decapitated, skull opened, and subsequently the cochlea removed from the temporal bone and transferred into ice-cold phosphate-buffered saline (PBS). Under a stereomicroscope (Leica-MZ6) and by means of fine forceps (Dumont), the cochlea was opened by removing the outer shell. In the next step, the spiral ligament, organ of Corti, and spiral ganglion were disconnected from the modiolus before the spiral ganglion was separated from the other parts and transferred to ice–cold Hank's buffered salt solution (HBSS) without calcium and magnesium. The spiral ganglia were enzymatically and mechanically dissociated. They were transferred to HBSS solution containing 0.1% trypsin (BIOCHROM, Berlin, Germany), 0.1% DNase I (ROCHE, Basel, Switzerland), and 0.01% collagenase and then incubated for 17–20 min at 37°C, 5% CO_2_, and 95% humidity. Dissociation was stopped by adding pre-warmed fetal calf serum, and the cells were transferred to a serum-free medium. Mechanical dissociation was performed by gentle trituration. Dissociated cells were then transferred into fibroblast specific medium containing 89% DMEM + L-glutamine, 10% FCS, and 1% penicillin/streptomycin.

### Cell Culture

Spiral ganglion cells were seeded at densities of 3 × 10^5^ cells per T25 culture flask, kept at 37°C, 5% CO_2_, and 95% humidity. The medium was exchanged every 2–4 days. When the cells reached confluence, they were detached by the addition of trypsin (concentrations between 0.05 and 0.25%)/0.02% EDTA solution. To stop the enzyme action, 3–5 ml pre-warmed medium with FCS was added. Cells were counted in a Neubauer counting chamber after adding 10 μl trypan blue (0.5%) to 10 μl of cell suspension. An overview of the different conditions used to prepare the cells for sorting is provided in Table 1.

**Table 1:** Overview on conditions for the different cell preparations.

**Preparation/sort number**	**Age of the animals [d]**	**Time to subcultivation [d]**	**Confluence in T25 [%]**	**Amount of trypsin [%]**	**Duration of trypsin incubation [min]**	**Harvested cells [%]**
1	3–4	8	100	0.05	7	70
2	3	6	90	0.05	7	80
3	2	4	90	0.05	7	90
4	2	4	70	0.25	5	95
5	3–4	8	100	0.05	7	70
6	1	6	100	0.05	7	70
7	3	5	95	0.15	4	90
8	2–4	5	100	0.15	4	90
9	3	8	100	0.15	4	95
10	2–3	11	100	0.15	4	95
11	2–4	8	100	0.15	4	95
12	2–4	9	100	0.15	4	95
13	2–4	7	100	0.15	4	95

### Immunolabeling

For immunolabeling, 1 × 10^4^ cells were seeded on coverslips positioned in 24-well plates and fixed after 72 h. Cells on coverslips were washed twice with PBS before fixation by the addition of 300 μl PFA (4%) to the wells for 10 min. After the removal of PFA, cells were again washed twice with Tris-buffered saline (TBS) and additionally incubated with TBS for 5 min. Then TBS was removed, 600 μl PBS was added to each well, and the cells were stored at 4°C until staining.

**Table:** For indirect immunocytochemistry, the following primary antibodies were used.

**Antibody against**	**Type**	**Source**	**Catalog-number**	**Dilution**
**Intracellular:**				
200kD-NF	polyclonal, chicken-IgY	Abcam, Cambridge, UK	Ab4680	1:10,000
S100, *astrocyte marker*	polyclonal, rabbit-IgG	Abcam	Ab868	1:100
Vimentin, *clone V9*	monoclonal, mouse-IgG	DakoCytomation, Wiesentheid, Germany	M0725	1:75
*carbonic-anhydrase II*	polyclonal, rabbit-IgG	Abcam	Ab6621	1:50
Connexin-26 (Cx26)	monoclonal, mouse-IgG	Invitrogen, Karlsruhe, Germany	13-8100	1:50
S100	polyclonal, rabbit-IgG	Sigma-Aldrich GmbH, Steinheim, Germany	S2644	1:100
**Extracellular:**				
Thy1.1, *clone Ox-7*	monoclonal, mouse-IgG	Merck Millipore, Darmstadt, Germany	MAB 1406	1:100
p75NGFR	polyclonal, rabbit-IgG	Abcam	Ab8874	1:400
As secondary antibodies were used:				
rabbit-IgG, *Cy3*-*conjugated*	polyclonal, goat-IgG	JacksonImmunoResearch Laboratories, Hamburg, Germany	111-165-045	1:500
chicken-IgY, *DL488*-*conjugated*	polyclonal, goat-IgG	Abcam	Ab96947	1:250
mouse-IgG, *Cy5*-*conjugated*	polyclonal, goat-IgG	Abcam	Ab97037	1:250
Direct immunolabeling of the cells for sorting was performed using:				
Thy1 (CD90.1), *clone Ox-7, FITC*-*conjugated*	monoclonal, mouse-IgG	Merck Millipore	CBL1500F	10 μl/1 × 10^6^ cells
p75NGFR (192-IgG), *PE*-*conjugated*	monoclonal, mouse-IgG	Santa Cruz Biotechnology, Inc., Heidelberg, Germany	Sc - 71691	1 μg/1 × 10^6^ cells

Antibodies were diluted by adding a solution containing 10 ml PBS and 0.858 ml bovine serum albumin (BSA) solution (0.7 g BSA in 2 ml aqua dest). For intracellular staining, the cells were permeabilized by the addition of 300 μl of 0.1% PBT (0.5 g Triton X-100 in 500 ml PBS) per well for 5 min at room temperature (RT). After washing with PBS (3×), the cells were first incubated for 1 h at RT in 20 μl of the solution containing the primary antibody followed by further washing steps. Then incubation with 20 μl of the secondary antibody followed by washing in PBS was performed. The cells were then covered using ProLong-Gold-Antifade reagent with DAPI (Life Technologies GmbH, Darmstadt, Germany). For this, 5 μl of the reagent was added on a slide and the coverslip with the cells was put on it such that no air bubbles were entrapped. The slides were dried for at least 12 h and then stored at 4°C while keeping them in the dark.

### Cell Sorting

The harvested cells were counted and transferred to the buffer solution for cell sorting consisting of 12.5 ml PBS and 1.79 ml BSA solution. A total of 1 × 10^6^ cells was re-suspended in 1 ml buffer solution. Staining for Thy1 (FITC-conjugated, fibroblasts) or p75NGFR (PE-conjugated, glial cells) was done at the concentrations recommended by the manufacturers by the addition of 10 μl Thy1-antibody and 20 μl p75NGFR antibody per 100 μl buffer solution with cells for 30 min and subsequent washing of the cell preparation. In addition, at least 5 × 10^4^ cells remained unlabeled as the negative control.

Cell sorting was done using a MoFlo^TM^-XDP Upgrade high-speed sorter (Beckman Coulter GmbH, Krefeld, Germany) equipped with a 488-nm solid-state laser. Before the sort, cells were filtered through a 70-μm nylon filter. The nozzle used had a diameter of 100 μm. The process was controlled and the data was analyzed by Summit-software 5.1.0, which is running the system. The negative controls were always investigated first to check and optimize settings.

To confirm the purity of each preparation, the collected fibroblasts were re-analyzed. Finally, collected fibroblasts were centrifuged at 800 rpm for 5 min, the supernatant removed, and the cells re-suspended in 5 ml warm medium before culturing the cells again.

### Microscopy

Immunolabeled cells were documented using an Olympus-Bx51 fluorescence microscope (Olympus, Hamburg, Germany) equipped with 4×, 10×, 20×, and 40× optics and the following filter sets: F41-017 (DL488), U-MWIG3 (Cy3), U-M41024 (Cy5), and U-MWU2 (DAPI). Image acquisition was done via a cooled color camera (CAM-XC10) and a 1.4 MPixel CCD chip using CellSense software (Olympus), which controlled all the settings for image acquisition. Individual settings are provided in the figure legends.

### Cryoconservation

After subcultivation to 80% confluence in T75 flasks, the cells were trypsinized for 6 min by the addition of 3 ml of 0.05% trypsin solution. The process was stopped by adding 10 ml of pre-warmed medium. The cell suspension was transferred to a 15-ml Falcon tube and the number of cells were determined. After centrifugation at 800 rpm for 4 min, the cell pellet was resuspended in a freezing medium (95% FCS, 5% DMSO) such that a cell concentration of 1.4 × 10^6^ cells per ml was achieved. Cells were then transferred into cryovials at 1-ml aliquots and frozen at −80°C. Two days later, the vials were stored in liquid nitrogen at −196°C.

After 3 weeks, cells were thawed again, taken into cell culture, and further subcultivated.

## Results

**Figure 1:**
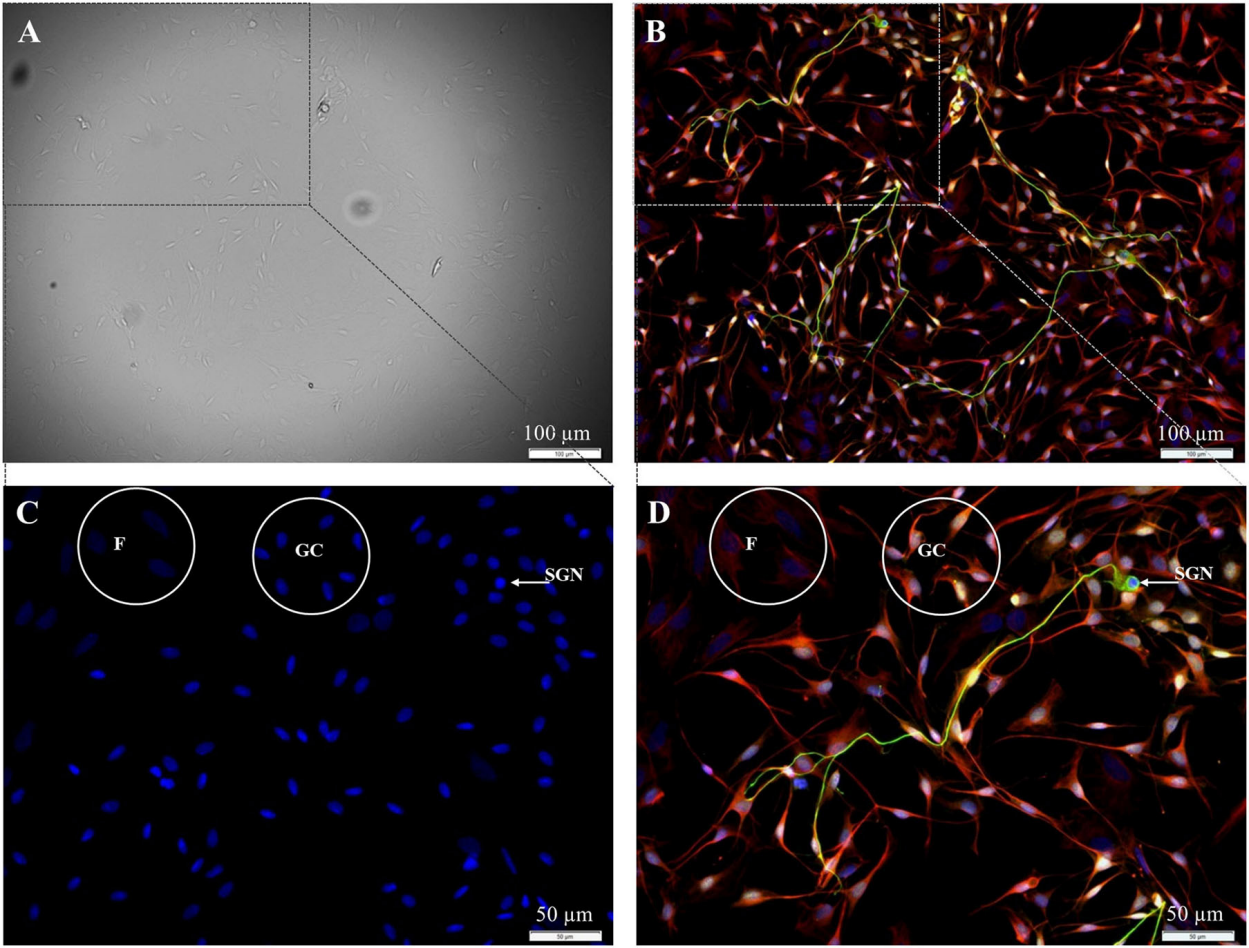
Isolated cells from the spiral ganglion after 72 h cultivation. **(A)** DIC-image, exposure time 40 ms. **(B–D)** Fluorescence images: 200 kD-NF (DL488, green), S100B-protein (Cy3, yellow), vimentin, c*lone V9* (Cy5, red), DAPI (blue). Exposure times: **(B)** DL488 1 s, Cy3 500 ms, Cy5 500 ms, DAPI 800 ms. **(C,D)** Higher magnification of the marked section in B. DL488 1 s, Cy3 200 ms, Cy5 1 s, DAPI 800 ms. **SGN:** 200kD-NF-positive spiral ganglion neuron. **GC:** S100B- and vimentin-positive glial cells. **F:** Vimentin-positive fibroblasts.

In the first step, the preparation of spiral ganglion cells was modified in a way that the fibroblast-specific medium was used for all preparation steps. Using this medium, SGN, glial cells, and fibroblasts were still found in the culture after 72 h of cultivation (Figure 1).

**Figure 2:**
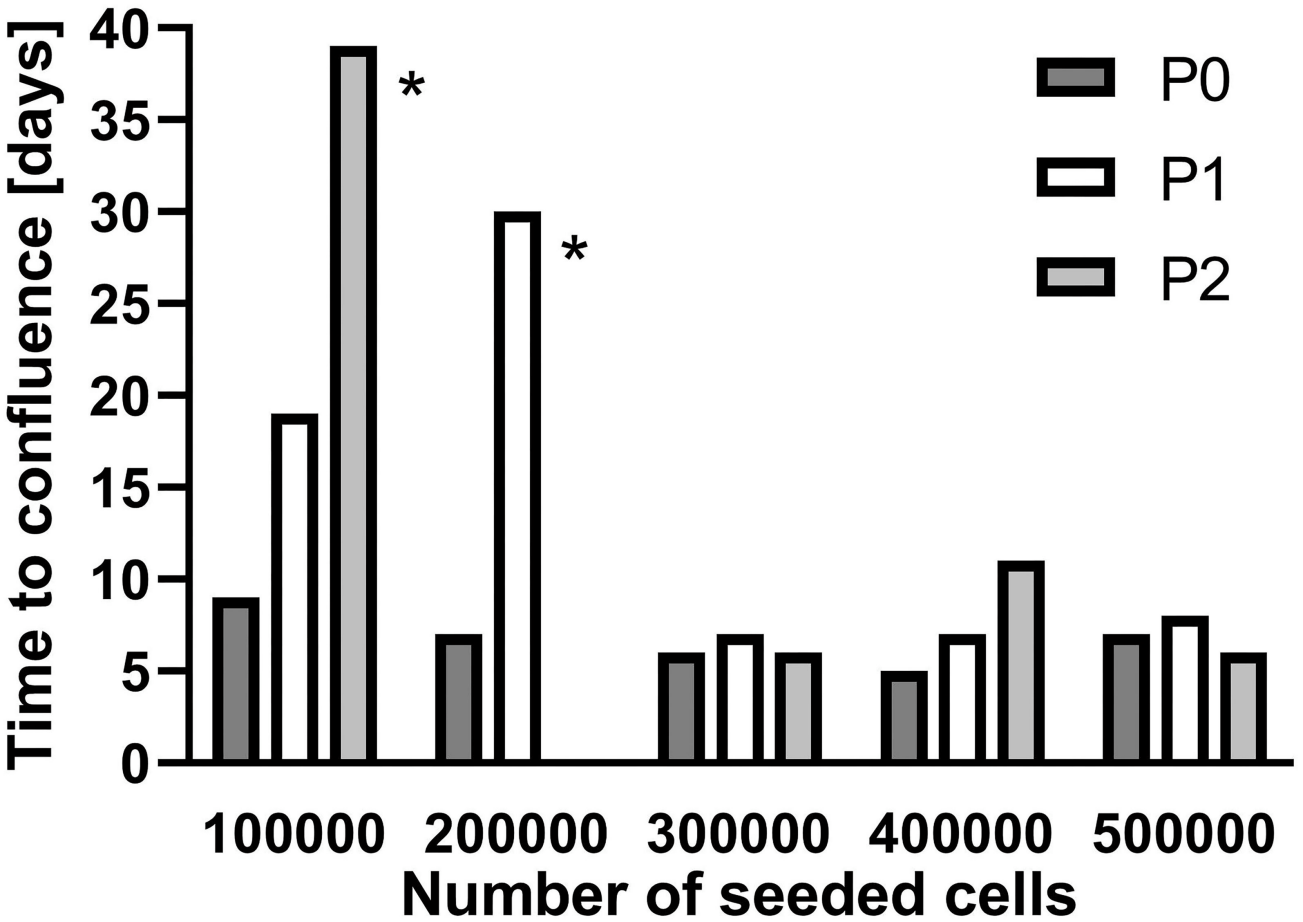
Subcultivation of isolated spiral ganglion cells in T25 flasks. Growth until confluence is provided in days for different seeded cell numbers. **P0:** Primary cell culture. **P1:** First passage. **P2:** Second passage. *subcultivated at 60–65% coverage.

To increase the number of available cells, SGC were seeded in culture flasks (25 cm^2^) at densities of 1 × 10^5^ to 5 × 10^5^ cells and subcultivated after confluence was achieved. Starting with 3 × 10^5^ cells, the time until confluence remained constant from passage P0 to P2 whereas for lower cell densities more time was necessary with each subcultivation to reach confluence (Figure 2). For all further subcultivation steps, a cell density of 3 × 10^5^ cells per flask was used.

**Figure 3:**
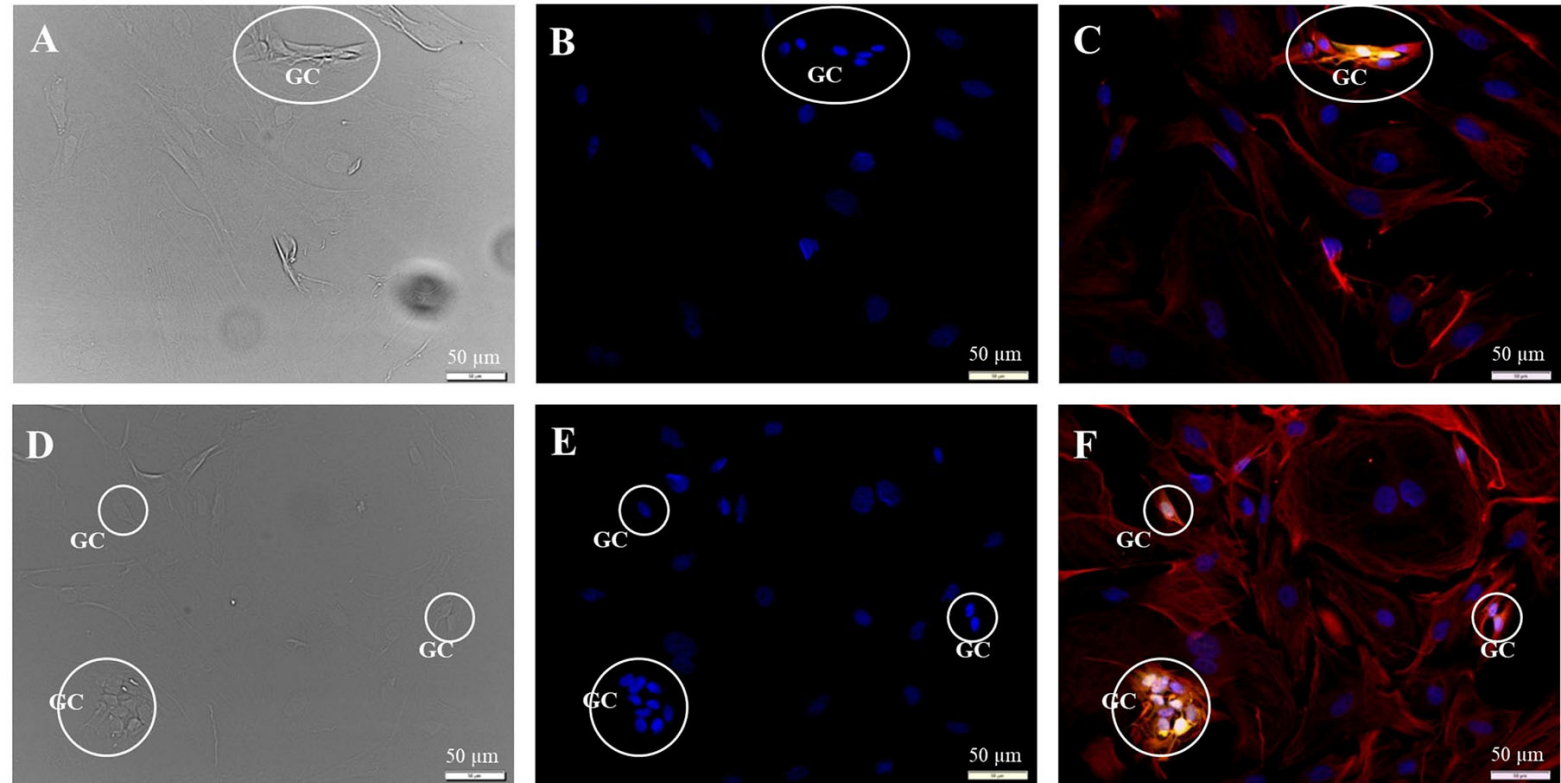
Subcultivation of the SGC culture in fibroblast specific medium 72 h after seeding. Fluorescence images: 200kD-NF (DL488, green), S100B-protein (Cy3, yellow), vimentin, c*lone V9* (Cy5, red), DAPI (blue). **(A–C)** Passage P1, exposure times: DIC 100 ms, DAPI 1 s, DL488 40 ms, Cy5 1 s, Cy3 50 ms. **(D–F)** Passage P2, exposure times: DIC 100 ms, DAPI 1 s, DL488 50 ms, Cy5 1 s, Cy3 137 ms. **GC:** S100B- and vimentin-positive glial cells.

Subcultivation of the cell preparation removed the neuronal cells, and the number of glial cells in the preparations was reduced to about 30%. Additionally, the distribution of cells within the cultures appeared altered. Glial cells seem to arrange in clusters, whereas fibroblasts are widely spread (Figure 3). These results were independent of the amount of trypsin used and the duration of incubation with trypsin.

**Figure 4:**
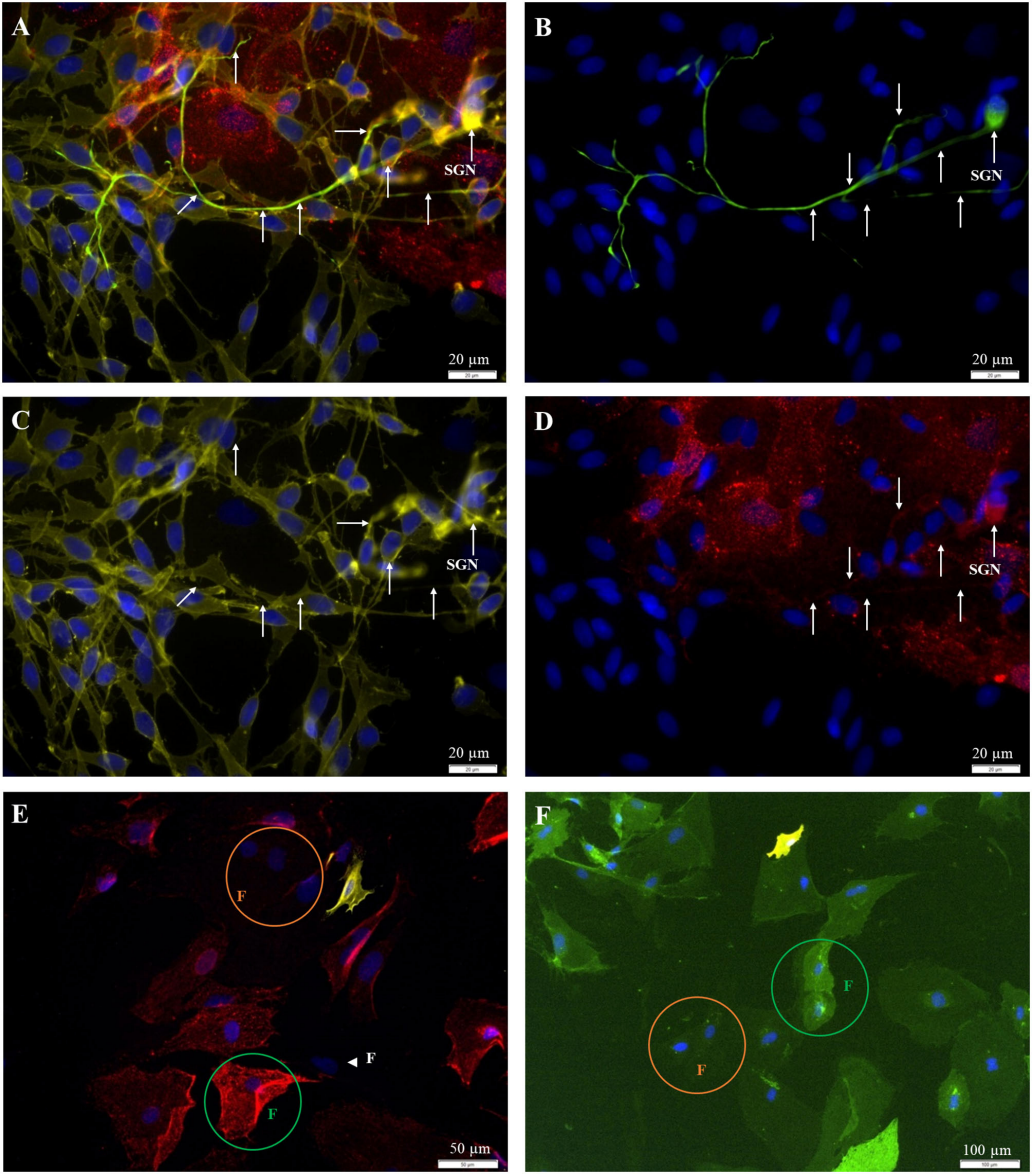
Fluorescent staining of cultures [**(A**–**D)** P0; **(E,F)** P2]. **(A)** 200kD-NF (DL488, green, exposure time: 1 s), Thy1.1, c*lone Ox-7* (Cy5, red, 5 s), p75NGFR (Cy3, yellow, 250 ms), DAPI (blue, 500 ms). **(B)** 200kD-NF of the SGN **(C)** p75NGFR-receptor (glial cells). **(D)** Thy1-protein (fibroblasts). **(E)** Indirect staining: Thy1.1, *clone Ox-7* (Cy5, red, 5 s), p75NGFR (Cy3, yellow, 600 ms), DAPI (blue, 1 s). **(F)** Direct staining: Thy1 (CD90.1), *clone Ox-7, FITC-conjugated* (green, 1 s), p75NGFR (192-IgG), *PE*-*conjugated* (yellow, 1 s), DAPI- (blue, 1 s). **White arrows:** SGN with neurites. **Green circle:** Strong Thy1-fluorescence. **Orange circle:** weak Thy1-fluorescence, **Arrowhead:** p75NGFR- und Thy1-negative cell with fibroblast-like morphology.

With this approach, only a fibroblast-enriched culture was achieved, and cells were now prepared for fluorescence-based cell sorting. To distinguish between neurons, fibroblasts, and glial cells, indirect labeling with fluorescent antibodies against the p75NGF receptor (glial cells), 200 kD neurofilament (SGN), and Thy1-glycoprotein (fibroblasts) was established with P0 preparations (Figures 4A–D) and later applied to P2 preparations (Figure 4E). Thy1 and p75NGFR were then used for direct immunofluorescence labeling of vital cells, which enabled cell sorting (Figure 4F). The neuron in Figure 4 appears to be stained not only for the 200 kD neurofilament but also for p75NGFR and Thy1. Thy1 positive fibroblasts can have either a weak or intense staining (Figures 4D–F). Additionally, few cells without staining were found (Figure 4E, white arrowhead). As the neurons were removed by subcultivation, cells were either positive for Thy1 (fibroblasts) or p75NGFR (glial cells), but no coexpression was detected.

**Figure 5:**
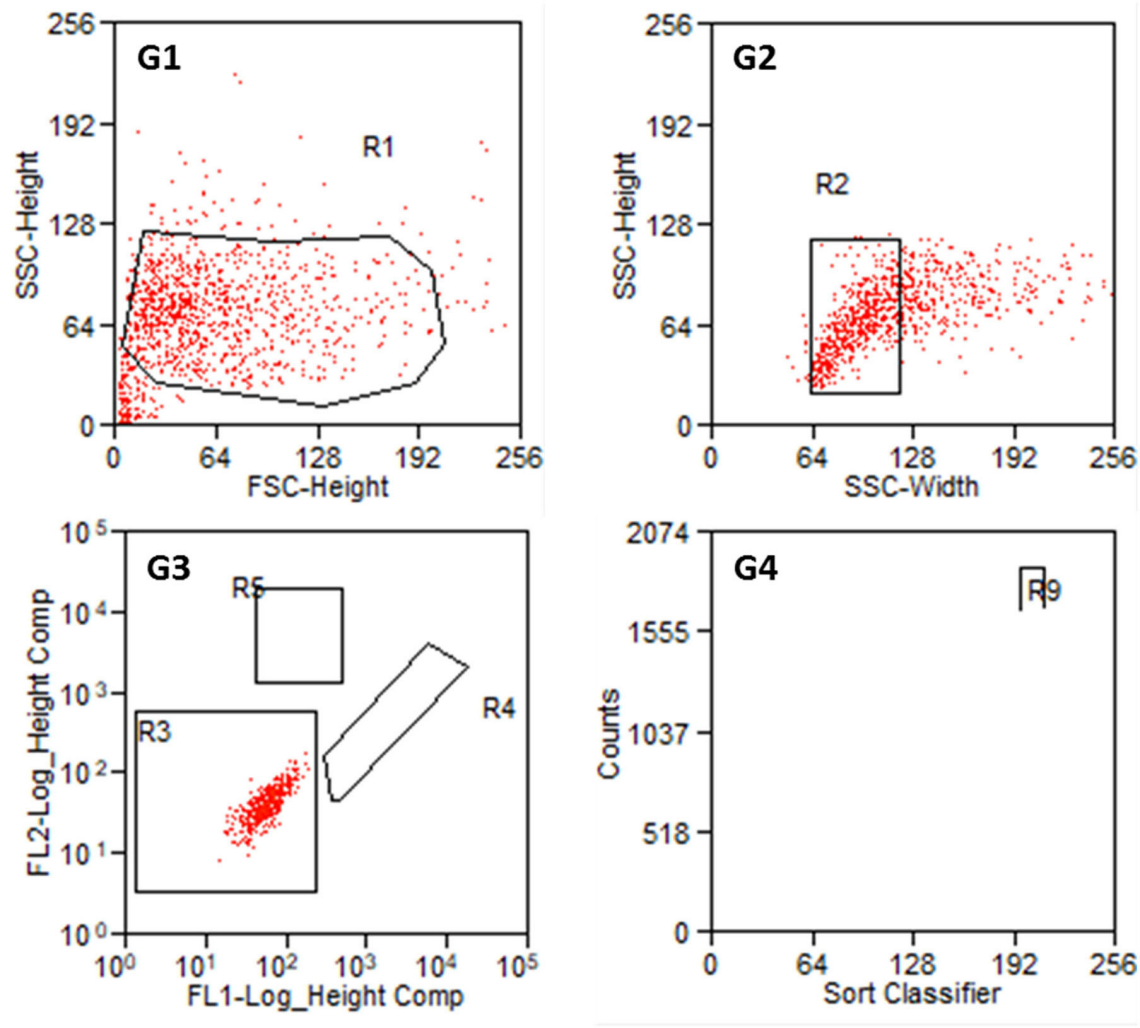
Results of the sorting process with non-labeled cells from P2. **G1:** Entire cell population in side scatter (SSC-Height) and forward scatter (FSC-Height). R1 is set to exclude cell debris. **G2:** Cell population from R1 in SSC (Width and Heights). **G3:** Autofluorescence of the cell population in R2. FL1 serves as FITC-channel (green) and FL2 as PE-channel (yellow). R3 is set such that FL1- values below 10^2.3^ and FL2-values below 10^2.7^ are taken as autofluorescence. R4: FITC positive cells. R5: PE positive cells. **G4:** Counts of detected cells within R4 and R5. R9 contains Thy1-FITC positive fibroblasts.

From each cell preparation, a negative control was tested first and the gates were defined. The first gate should remove cell debris and cell cluster. In gate 3, cells were classified according to their autofluorescence (Figure 5). Regions R4 (FITC-fluorescence) and R5 (PE) should still be empty. Intensity values below 10^2.3^ (FITC-channel) and below 10^2.7^ (PE-channel) were defined as autofluorescence. Investigating negative controls, no counts were detected in R4 and R5 for all the samples.

**Figure 6:**
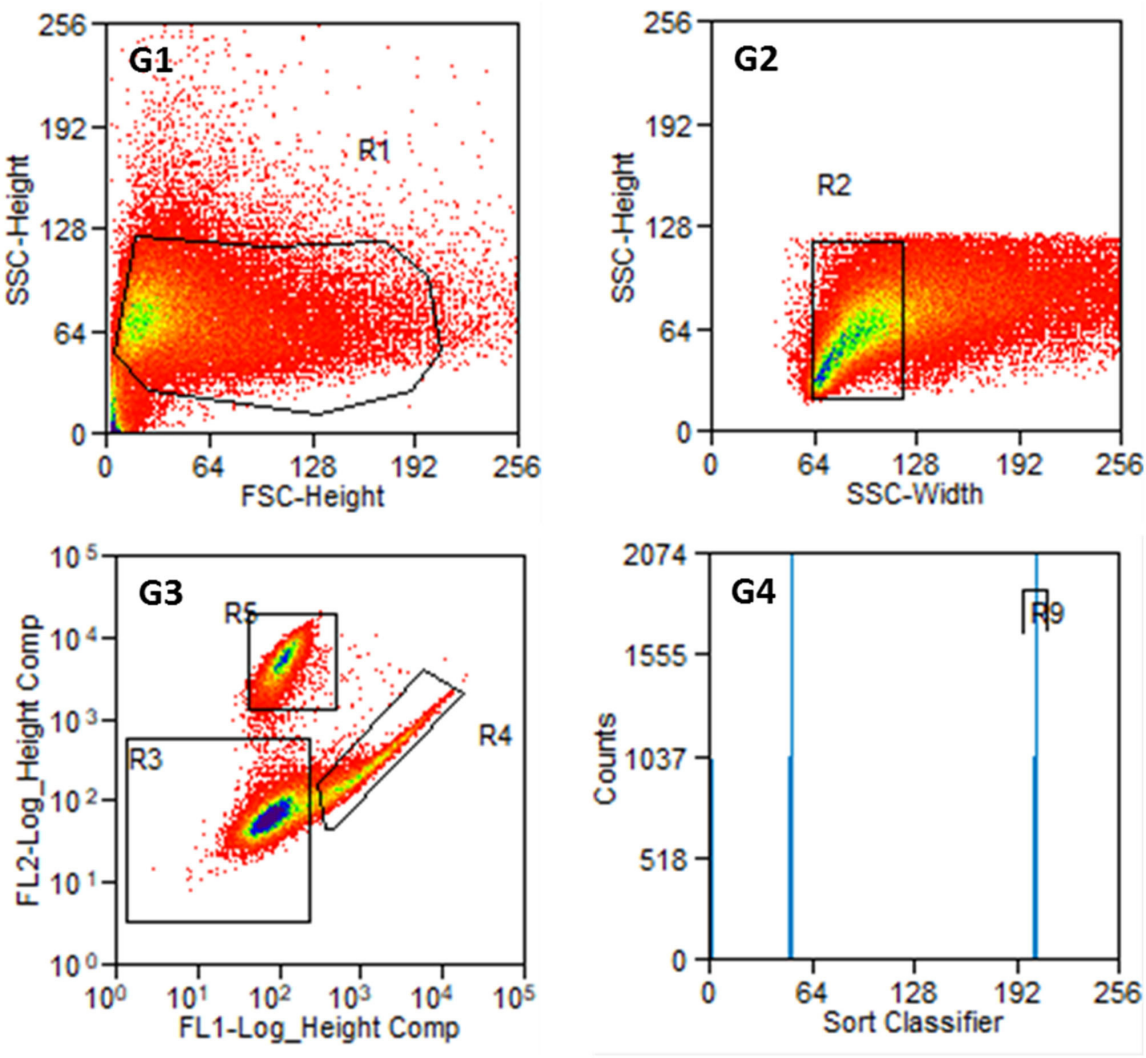
Results of the sorting process with labeled cells from P2. **G1:** Entire cell population in side scatter (SSC-Height) and forward scatter (FSC-Height). **G2:** Cell population from R1 in SSC (width and heights). **G3:** Cell population from R2 according to their fluorescence signal. R3: non-labeled or weakly stained cells, R4: Thy1-FITC-positive fibroblasts, R5: p75NGFR-PE-positive glial cells. **G4:** Cell counts from R4 and R5 with R9 containing Thy1-FITC positive fibroblasts. The number of cells at distinct points decreases from blue to red in G1 to G3.

An example of the results of a stained preparation is presented in Figure 6, where cells were also found in R4 and R5. The number of counts in both regions is given in G4 with the R9 region comprising all cells counted in R4, which are the FITC-labeled fibroblasts.

**Figure 7:**
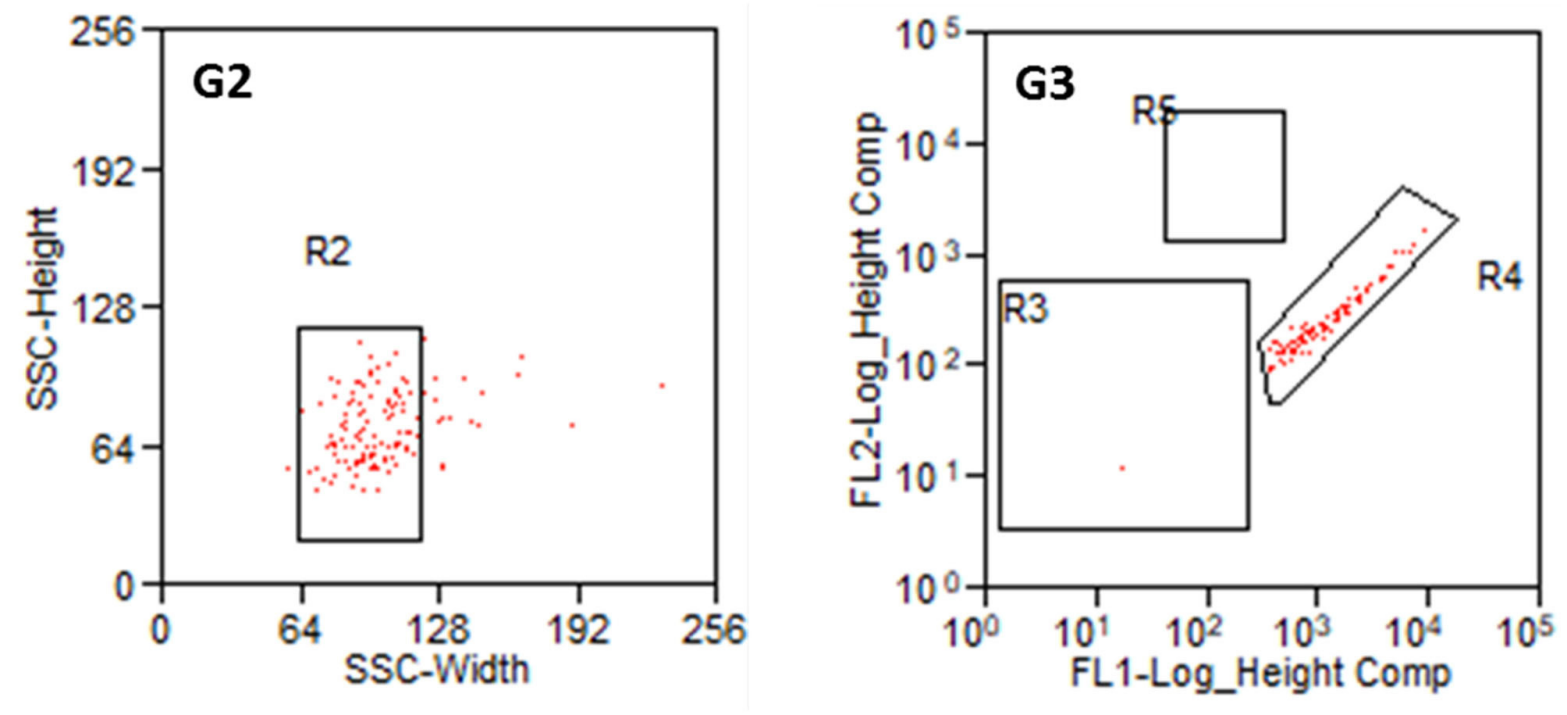
Reanalysis of collected fibroblasts. **G2:** Cell population from R1 in SSC (Width and Heights). **G3:** Cell population from R2 according to their fluorescence signal. R3: non-labeled or weakly stained cells, R4: Thy1-FITC-positive fibroblasts, R5: p75NGFR-PE-positive glial cells.

Parts of the collected fibroblasts were re-analyzed (Figure 7). This confirmed a very high purity of fibroblasts. Only in two out of 13 preparations, a single cell was detected in R5 (glial cells). In all other preparations, only fibroblasts (R4) were detected. This results in a purity of 99.9% cochlear fibroblasts, when averaged over all preparations. Some cells were damaged during the sorting procedure in all experiments.

**Figure 8:**
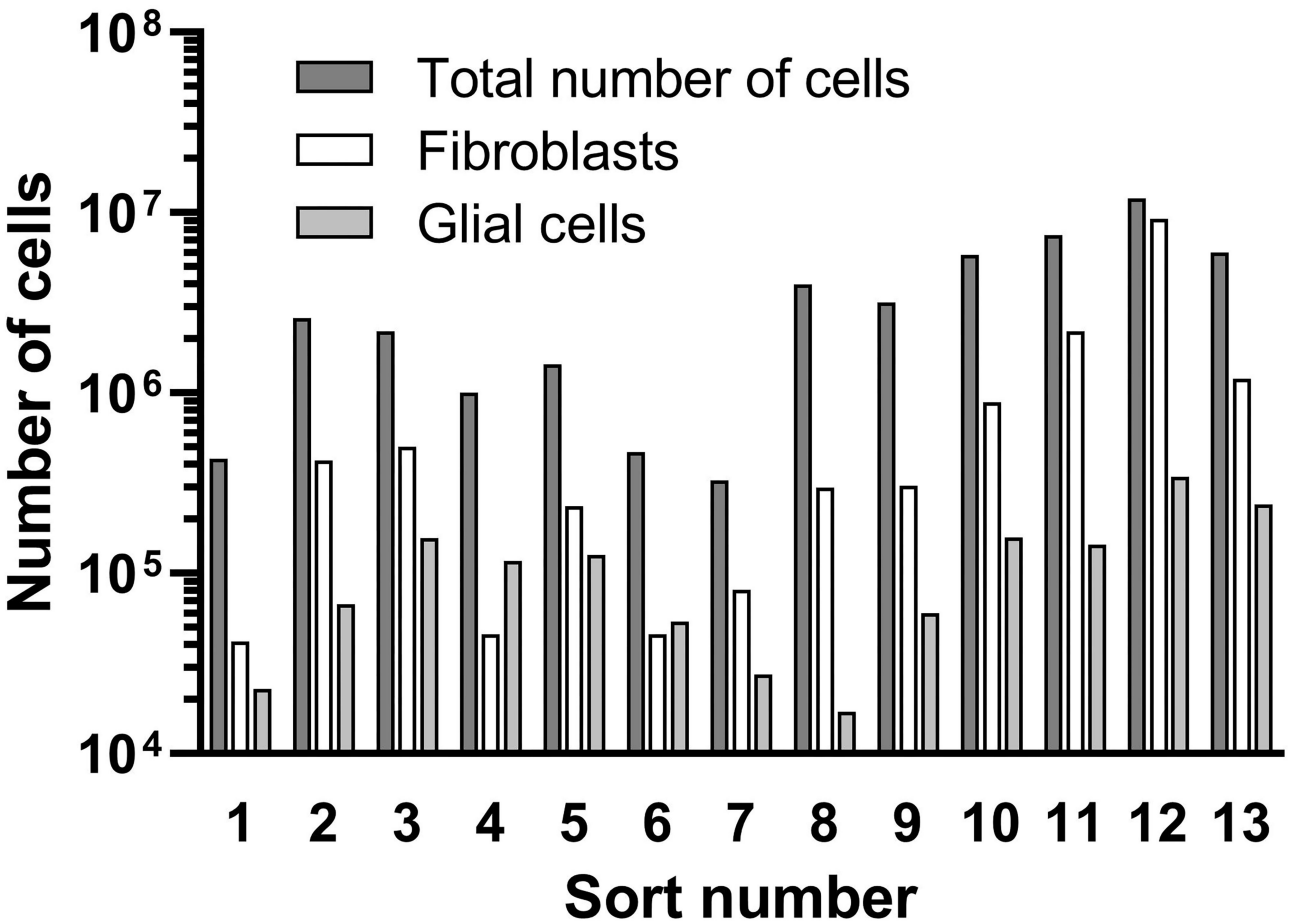
Results of the cell sorting procedure. Total cell numbers, received fibroblasts and glial cells are presented.

An overview of the counted cell numbers for all preparations is provided in Figure 8. The total number of counted cells was between 3.3 × 10^5^ (prep 5) and 1.2 × 10^7^ (prep 12) with an average number of 3.6 × 10^6^ ± 3.3 × 10^6^ cells (mean ± SD). On average, the number of collected fibroblasts was 1.2 × 10^6^, which is 33% of the total cell number before the sorting process. The number of counted glial cells was 1.2 × 10^5^ (3.3% of total cells), far lower than the fibroblasts. Only in preparations 4 and 6 more glial cells than fibroblasts were detected.

**Figure 9:**
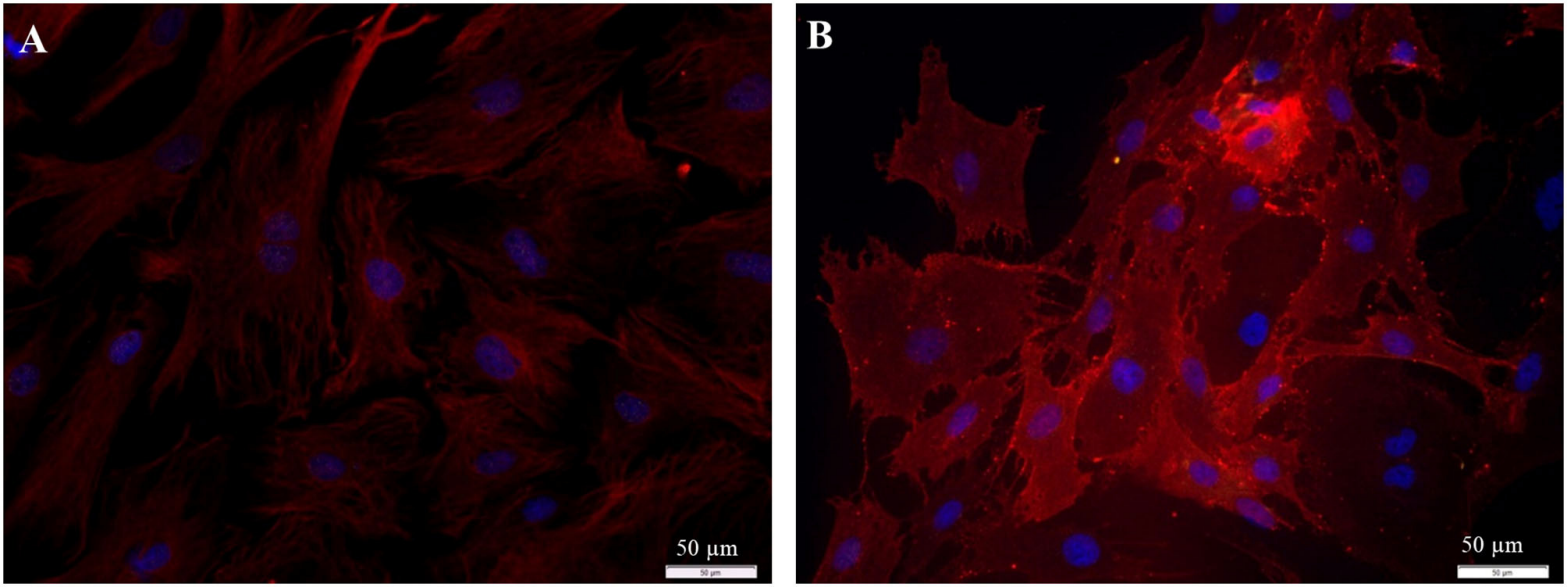
Staining of collected fibroblasts after the sorting process (P3 after 72 h cultivation). **(A)** Intracellular staining: vimentin, *clone V9* (Cy5, red, 5 s), S100B-protein (Cy3, yellow, 100 ms), DAPI (blue, 1 s). **(B)** Staining of surface proteins: Thy1.1, *clone Ox-7* (Cy5, red, 1 s), p75NGFR (Cy3, yellow, 1 s), DAPI (blue, 1 s).

The collected fibroblasts were cultured and subcultivated again. Intracellular staining revealed that all cells (100%) were Vimentin-positive and S100B negative (Figure 9A), indicating that (nearly) only fibroblasts were in the culture. This was confirmed by staining for p75NGFR and Thy1, where only Thy1 fluorescence was detected (Figure 9B). Additionally, all cells showed the typical morphology of fibroblasts. Again, cells with strong and weak fluorescence signals were detected.

The received fibroblast preparations were subcultivated for up to 16 passages. A relatively slow growth of cells was observed between P3 and P5 followed by P6 to P8 with the highest proliferation rates. Starting with P9 or P10, cell growth slowed down again before it stopped between P13 and P16. First morphological changes (granules were observed) could be observed starting from P5, which became more pronounced (nuclei appeared homogenous) around P10.

A first attempt was made for cryoconservation of the received cochlear fibroblast preparations (P5). After 3 weeks, cells were thawed and subcultivated for at least three more passages. After 24 h, about 80% of the cells adhered to the surface and after 3 days of culture, no differences to the non-frozen fibroblasts were found morphologically. Only the growth rate was slightly diminished from P2 after thawing.

**Figure 10:**
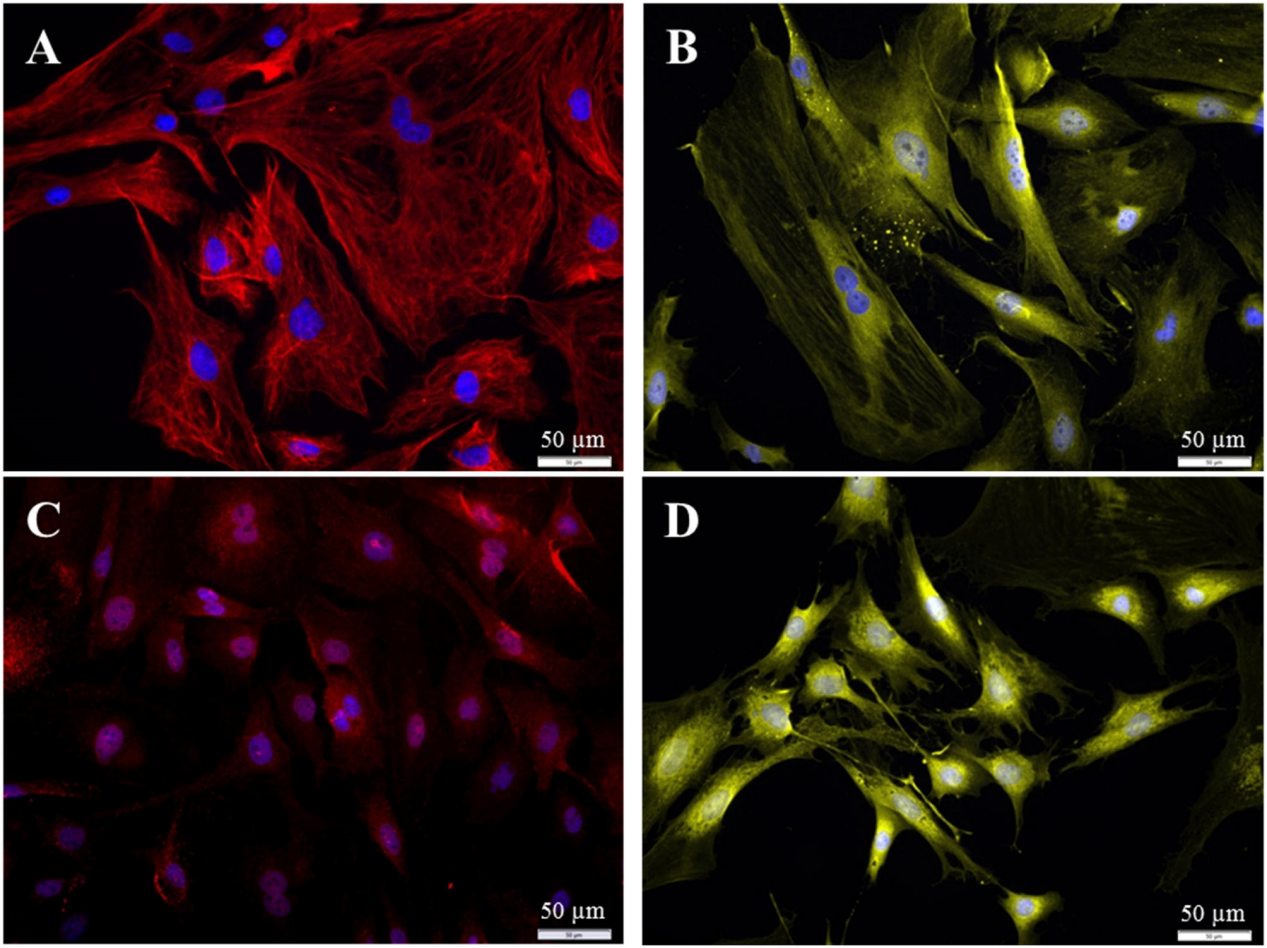
Immuncytochemical characterization of the purified fibroblasts (P3 after 72 h cultivation). **(A)** *Anti-Vimentin, clone V9* (Cy5, red, 3 s). **(B)** *Anti-S100* (Cy3, yellow, 400 ms). **(C)** *Anti-Cx26* (Cy5, red, 5 s). **(D)** *Anti-carbonic-anhydrase II* (Cy3, yellow, 100 ms). All preparations were additionally stained for DAPI (blue, 1 s).

Different populations of fibroblasts from the spiral ligament were distinguished according to their immunhistochemical profile (18–23). To be able to compare our fibroblasts from Rosenthal's canal with these published results, additional staining against S100, Connexin-26, and carbonic anhydrase II was performed. The fibroblasts from the current study were found to be positive for S100, vimentin, CAII, and Cx-26 (Figure 10).

## Discussion

Freshly prepared spiral ganglion cells (SGC) are typically used to investigate the effects of drugs or electrical stimulation on spiral ganglion neurons (SGN) (12, 13). When addressing the growth of fibrous tissue in *in vitro* experiments, immortalized cells lines, such as NIH/3T3 or L929, are used (24). Here, it might be beneficial to have organ-specific primary fibroblasts available. When preparing the SGC culture, apart from spiral ganglion neurons, glial cells and fibroblasts are also part of the culture (25, 26). Based on the SGC culture, enrichment and purification of Schwann cells has already been described (15, 16). Therefore, in the current study, we isolated fibroblasts from the SGC culture.

When establishing the staining protocol for our SGC preparation, it was first observed that all 200-kD neurofilament negative cells were positive for vimentin but no neurons expressed vimentin. Studying the human spiral ganglion (SG) (27) and the SG of 3–4 months old Wistar rats (28), all neurons were negative for vimentin. Furthermore, for cells from the spinal ganglion and the Ischias nerve of 10-week-old rats, it was documented that glial cells in the PNS and fibroblasts express vimentin (29), which is in accordance with the current results. The 200-kD neurofilament could only be detected in type II SGN (27, 30) when using mature cochleae, whereas in early developmental stages it is found in type I and type II neurons (31). Therefore, we can expect that in our preparations from postnatal days 2–4 both types of neurons are detected. Additional staining against the glial cell-specific S100B protein revealed that some of the vimentin-positive cells were also positive for the S100B protein. As Schwann cells are the main glial cells in the PNS (25, 32, 33), we expect that the S100B positive cells are mainly Schwann cells. But also satellite cells were described as vimentin- and S100-positive (28, 29, 34). Van Neerven et al. (29) reported that fibroblasts in the PNS were S100B negative and vimentin-positive whereas Schwann cells and satellite cells were S100B- and vimentin-positive. Regarding the SGC, more than 50% of the non-neural cells in the culture were other cells than glial (Schwann) cells (16). In our study, more glial cells than fibroblasts were found in P0 preparations, and only after subcultivation, the number of fibroblasts increased to more than 50 %.

In contrast to the published procedure of preparing SGC (12), a fibroblast-specific medium was used in the current study. Using DMEM plus FCS should enhance the proliferation rate of fibroblasts but not glial cells (25, 35). As other authors reported that after 72 h the proliferation rate of fibroblasts was enhanced whereas after 48 h Schwann cells were still outnumbering the fibroblasts; cells were cultured on coverslips for 72 h. Before seeding the cells (except for P0), cells grew in culture flasks and had to be trypsinized. Incubation in 0.25% trypsin 0.02% EDTA for 4 min resulted in the detachment of 100% of the cells. Already after the first passage no SGN was observed and the number of glial cells was reduced to about 20–30% in the culture. The reduction in glial cells could be explained by the lack of SGN (16) or the use of a fibroblast-specific medium (35).

To separate fibroblasts and glial cells by cell sorting, immunolabeling of the cells has to be done using extracellular marker proteins. From other studies, it was known that fibroblasts express the surface protein Thy1 (CD90) and glial cells the p75NGF receptor (p75NGFR) (33, 36, 37). For the spiral ganglion, neurons and fibroblasts were shown to be Thy1 positive (38, 39). Furthermore, satellite cells and neurons express p75NGFR (40). Staining of the p75NGFR was also used die purify Schwann cells from the spiral ganglion (16). These authors additionally report that some neurons were also collected using their approach. These findings perfectly match with the immunolabeling pattern in the current study where non-neuronal cells were either Thy1- or p75NGFR-positive and neurons were stained for both the proteins. As discussed above, neuronal cells were removed from the culture after the first passage. Therefore, we can be sure to have only fibroblasts and glial cells in our preparations for cell sorting when using cells of the second passage.

Altogether, 13 preparations were used for cell sorting, some of them with slightly modified protocols (compare Table 1). All the collected Thy1 positive fractions were re-analyzed. Some counts were always outside the regions defined for fibroblasts and glial cells. This could potentially be explained by a reduced fluorescence intensity due to first exposure to the LASER (41) or some (mechanical) damage to the cells during the process. More than 99% purity of the collected fibroblasts, as determined by the device, was confirmed while immunolabeling the cells after the sort. Nearly no glial cells were detected at P3. In most preparations, the number of counted fibroblasts was relatively low compared to the original cell number. On average, 74.5% of the cells were lost during the procedure. This is larger than described for the separation of Schwann cells and fibroblasts using magnetic-activated cell sorting (35) for cells from rat sciatic nerve. One of the reasons for this might be the settings used for the sorting process. To receive a fibroblast preparation as pure as possible it might be that with the settings chosen weaker labeled cells were not detected. In all of our preparations, strong and weakly labeled cells were found. This observation was already described by Fields et al. (38) for antibodies against the Thy1 protein. It could also be that some unlabeled cells were still in the culture as macrophages are described for the PNS (42), and these cells were negative for Thy1. Furthermore, it is also known that fibroblasts are a heterogeneous group within a population and not all sub-groups might be Thy1 positive (43). The latter explanation appears to be unlikely in our investigation as nearly no unlabeled cells were detected before cell sorting.

As the ratio of counted fibroblasts and glial cells differed between preparations, some damage to the epitopes during trypsinization cannot be excluded, especially with long incubation times or high concentrations (44). With 0.25% trypsin in the solution, the total loss of cells was with 83.7% very high, and much more glial cells than fibroblasts were counted. Using only 0.05% trypsin in the solution, which is the standard for weakly adherent cells (44), cell loss was reduced and the number of fibroblasts increased. Here, maybe not all fibroblasts were detached from the culture flasks. Best results were achieved with 0.15% trypsin in the solution. Using this approach, cell loss was about 60%, which is in the range of the reported cell loss for magnetic-activated cell sorting (35). Besides preparation 4 (high trypsin), there was one more preparation with more counted glial cells than fibroblasts. This preparation (sort 6) was the only preparation where the animals were only 1 day old instead of 2–4 days. This might be the reason for the different result in this preparation.

Compared to immortalized cell lines, primary cells and their subcultures have the advantage of keeping most characteristics from their host tissue, also *in vitro* (3). The purified cochlear fibroblasts were further subcultivated until P16. The observed changes in cell growth correspond to the phases described by Hayflick and Moorhead (45). Phase I (Lag-phase) is the primary cell culture until a confluent monolayer is reached. This is followed by phase II (Log-phase) with good proliferation rates and phase III with a decreasing proliferation rate and finally death of the cell culture. The cochlear fibroblasts seem to enter the latter phase after 9–10 passages, at least with the seeding density used in this study.

The purified fibroblasts were analyzed according to the immunohistochemical profile that is described for fibroblasts of the spiral ligament (18–23). They were positive not only for vimentin but also for carbonic anhydrase II, connexin 26, and the S100 protein. The expression pattern of the fibroblasts from Rosenthal's canal appears to be similar to that of type I fibroblasts of the spiral ligament, which can be found predominantly in the spiral ligament distal from scala tympani (11). Here, a more detailed characterization would be necessary.

Finally, and as not all cells might be suitable for cryoconservation, purified fibroblasts were frozen and thawed again. Cells with a survival rate of about 80% and an unchanged morphology are considered suitable for cryconservation (46). Therefore, we conclude that the described purified fibroblasts from Rosenthal's canal are also suitable for cryoconservation.

With the described procedure, fibroblasts could be purified from the SGC preparation and are now available for further *in vitro* experiments. As the cells can be subcultivated and frozen, cells from one preparation could be used for experiments for days and weeks without frequent new preparation from the animals.

## Data Availability Statement

The original contributions presented in the study are included in the article/supplementary material, further inquiries can be directed to the corresponding author/s.

## Ethics Statement

Ethical review and approval was not required for the animal study in accordance with the local legislation and institutional requirements.

## Author Contributions

AA: investigation, formal analysis, and writing—original draft. K-HE: methodology, validation, and writing—review and editing. TL: funding and writing—review and editing. GP: conceptualization, supervision, and writing—review and editing. All authors contributed to the article and approved the submitted version.

## Funding

This study was supported by BMBF Innovationswettbewerb Medizintechnik (FKZ: 13EZ1001B) and BMBF RESPONSE “Partnerschaft für Innovation in der Implantattechnologie” (FKZ 03ZZ0925A).

## Conflict of Interest

The authors declare that the research was conducted in the absence of any commercial or financial relationships that could be construed as a potential conflict of interest.

## Publisher's Note

All claims expressed in this article are solely those of the authors and do not necessarily represent those of their affiliated organizations, or those of the publisher, the editors and the reviewers. Any product that may be evaluated in this article, or claim that may be made by its manufacturer, is not guaranteed or endorsed by the publisher.
